# Ecological Momentary Assessment of Physical Activity: Validation Study

**DOI:** 10.2196/jmir.7602

**Published:** 2017-07-18

**Authors:** Gregory Knell, Kelley Pettee Gabriel, Michael S Businelle, Kerem Shuval, David W Wetter, Darla E Kendzor

**Affiliations:** ^1^ Michael and Susan Dell Center for Healthy Living Department of Health Promotion and Behavioral Sciences University of Texas Health Science Center (UTHealth) at Houston Houston, TX United States; ^2^ Michael and Susan Dell Center for Healthy Living Department of Epidemiology, Human Genetics & Environmental Sciences University of Texas Health Science Center (UTHealth) at Houston Austin, TX United States; ^3^ Department of Family and Preventive Medicine The University of Oklahoma Health Sciences Center Oklahoma City, OK United States; ^4^ American Cancer Society Department of Intramural Research Atlanta, GA United States; ^5^ Huntsman Cancer Institute Department of Population Health Sciences University of Utah Salt Lake City, UT United States

**Keywords:** accelerometry, behavioral risk factor surveillance system, ecological momentary assessment, self-report, data accuracy

## Abstract

**Background:**

Ecological momentary assessment (EMA) may elicit physical activity (PA) estimates that are less prone to bias than traditional self-report measures while providing context.

**Objectives:**

The objective of this study was to examine the convergent validity of EMA-assessed PA compared with accelerometry.

**Methods:**

The participants self-reported their PA using International Physical Activity Questionnaire (IPAQ) and Behavioral Risk Factor Surveillance System (BRFSS) and wore an accelerometer while completing daily EMAs (delivered through the mobile phone) for 7 days. Weekly summary estimates included sedentary time and moderate-, vigorous-, and moderate-to vigorous-intensity physical activity (MVPA). Spearman coefficients and Lin’s concordance correlation coefficients (LCC) examined the linear association and agreement for EMA and the questionnaires as compared with accelerometry.

**Results:**

Participants were aged 43.3 (SD 13.1) years, 51.7% (123/238) were African American, 74.8% (178/238) were overweight or obese, and 63.0% (150/238) were low income. The linear associations of EMA and traditional self-reports with accelerometer estimates were statistically significant (*P*<.05) for sedentary time (EMA: ρ=.16), moderate-intensity PA (EMA: ρ=.29; BRFSS: ρ=.17; IPAQ: ρ=.24), and MVPA (EMA: ρ=.31; BRFSS: ρ=.17; IPAQ: ρ=.20). Only EMA estimates of PA were statistically significant compared with accelerometer for agreement.

**Conclusions:**

The mobile EMA showed better correlation and agreement to accelerometer estimates than traditional self-report methods. These findings suggest that mobile EMA may be a practical alternative to accelerometers to assess PA in free-living settings.

## Introduction

Using self-report measures to assess physical activity (PA) can result in over-reporting of PA volume by respondents [1-3]. Possible explanations for this phenomenon include the respondent’s desire to be perceived as “active” (ie, social desirability bias) and cognitive challenges with accurately recalling PA duration, frequency, and/or intensity. Accelerometers are an attractive alternative to self-report measures because of their ability to directly capture accumulated ambulatory PA across several days of observation. Yet, accelerometers have limitations, including their inability to detect several common PA modes such as swimming, cycling, and muscle strengthening activities. Furthermore, accelerometers are not capable of providing contextual information pertinent to PA behavior, including the domain of PA (ie, leisure time, occupational, transit, domestic/housework), biomechanical and physiological demands/types of PA (eg, aerobic, anaerobic activity, flexibility training, and balance training), location where the PA occurred (eg, home, gym, and work), and whether the person engaged in PA alone or with a partner/group/trainer [4].

A less commonly utilized approach to PA assessment, the ecological momentary assessment (EMA), may overcome many of the limitations of traditional self-report measures while providing contextual information on PA. The EMA is an approach to measurement that allows individuals to repeatedly report on their experiences in real time, in real-world settings, over time, and across contexts (eg, mode, type, and location) [5]. This unique approach to PA assessment has the potential to minimize recall bias by allowing the respondent to report their activity as it occurs, or very shortly thereafter [6]. Short recall time frames are preferable because specific behaviors can be recalled using episodic memories rather than generic memories of past events that require estimation and computation strategies to assist recall [7,8]. The use of shorter recall time frames has been more extensive among diet- and time-use researchers but has more recently garnered attention among PA measurement researchers [9,10]. An additional concern with traditional self-report PA measures is the possibility of social desirability bias [1]. As is the case with recall bias, the impact of social desirability bias may be minimized in with shorter recall time frames [1]. Therefore, the shorter recall time frame characteristic of EMA may sufficiently minimize recall and social desirability bias.

EMA techniques may also help to bridge the gap between self-report and objective assessment of PA by addressing many of the major limitations of both options (social desirability and recall bias, void of contextual information). Research has demonstrated that EMA is a feasible tool for monitoring health behaviors among at-risk groups of adolescents and adults [11,12]. Previous work has also indicated that EMA protocols are adhered to by participants [13]. Although EMA has been shown to be a practical application for PA assessment, little is known about the validity of EMA for estimating recent time spent in PA across broad intensity categories. Such information is critically important when interpreting study findings within the context of current public health guidelines for PA [14].

Therefore, the primary objective of this study was to assess the correlation and agreement between daily diary EMAs of PA and accelerometer-derived estimates of PA among a group of free-living adults. The secondary purpose was to compare the agreement between EMA and accelerometer estimates of PA with traditional self-reported PA questionnaire estimates and accelerometer estimates. It was hypothesized that EMA estimates of PA and sedentary behavior would have acceptable convergent validity measurement properties and that EMA would display better agreement with accelerometer estimates than traditional self-reported PA from questionnaires.

## Methods

### Design Overview and Participants

This study is a secondary data analysis of the Pathways between Socioeconomic Status and Behavioral Cancer Risk Factors Study (PATHS). The PATHS was a 7-day prospective observational study designed to characterize proximal predictors of health behavior using mobile phone–based EMA. A subset of the PATHS participants also received a mobile phone–based sedentary behavior intervention (for details see Kendzor et al, 2016) [15]. A racially and ethnically diverse community sample of adults living in Dallas, Texas, the United States of America, were recruited for participation through print advertisements in local newspapers, advertising circulars, and flyers on The University of Texas Southwestern Medical Center campus and in the Dallas metropolitan area. A total of 248 participants were screened over the telephone. Of those, 238 (96%) met the eligibility criteria and were enrolled in the study. The study participants attended an in-person visit where they met with study staff and were (1) measured for height and weight, (2) asked to complete a questionnaire on demographics and various health behaviors (including PA and sedentary behavior), (3) provided a mobile phone, and (4) fitted with an accelerometer. The participants were instructed to carry the mobile phone and wear the accelerometer for 7 consecutive days following the initial visit. After the 7-day period, they were asked to return their accelerometer and mobile phone to the study site. Participants were compensated up to US $130 for the completion of all aspects of the study. The complete PATHS study methods are described elsewhere in greater detail (Kendzor et al, 2016 [15]). This study was approved by the institutional review boards of The University of Texas Southwestern Medical Center and The University of Texas Houston Health Science Center. Informed consent was obtained from all participants.

### Data Collection

#### Participant Characteristics

The participants completed questionnaires on laptops or tablet computers, including items on demographic and socioeconomic factors such as age, race and ethnicity, educational attainment, employment status, and income. Household income was classified as low if it was reported to fall between less than or equal to 100% of the 2012 Federal Poverty Threshold and 199% of Federal Poverty Threshold.

Weight and height were measured by study staff, and body mass index (BMI) was calculated using the standard formula (kg/m²).

#### Self-Reported Physical Activity and Sedentary Behavior Measures

Self-reported PA and sedentary behavior were measured using three instruments with varying periods of assessment: EMA, “past 24 hours”; 7 items from the 2007 Behavioral Risk Factor Surveillance System (BRFSS), “usual week”; and ten items from the 2011 International Physical Activity Questionnaire (IPAQ), “past 7 days.”

For the EMA, participants were provided with a LG Optimus T Smartphone with the Android 2.2 operating system, on which they were prompted to complete daily diary assessments of health behavior (including PA) over a 7-day observation period. The EMA program was developed by and accessed through the e-Health Technology resource provided by the Duncan Family Institute at the MD Anderson Cancer. Participants completed daily diary assessments once daily, 30 min after their self-reported usual wake time.

First, participants were asked to select the time spent over the past 24 hours in moderate-intensity PAs via 8 response options that ranged from 0 to ≥70 min, in increments of 10 min. Participants were provided with examples of moderate-intensity PAs (eg, brisk walking and bicycling) to aid with recall. Next, participants were asked to report the time spent per day in vigorous-intensity PAs. Again, examples of vigorous-intensity PAs (eg, running and aerobics) and 8 response options were provided to facilitate recall. Summary estimates were expressed as the mean value within response categories for moderate- and vigorous-intensity PA separately. For example, if the participant reported 20 to 29 min of moderate-intensity PA over the previous 24 hours, 24.5 min was used. These values were summed across all days of observation and expressed as time spent per week in moderate-, vigorous-, and moderate- to vigorous-intensity PA (MVPA).

As part of a larger survey administered to participants during the initial visit (ie, before EMA administrations and accelerometry), 7 items from the 2007 BRFSS PA questionnaire were utilized to assess usual leisure-time PA; these items were adapted for self-administration. Participants were asked to report the duration (minutes per day) and frequency (times per week) of PA within broad intensity categories (ie, moderate- and vigorous-intensity PA). The summary estimate reflecting moderate- to vigorous-intensity leisure-time PA was computed as the product of the reported duration and frequency (minutes per week) summed across intensity categories. The PA questions from BRFSS have previously been shown to be reliable and valid [16].

Ten items from the 2011 IPAQ were also included in the initial survey to assess occupational- and transportation-related PA in the past week [17]. For occupational PA, participants were asked to report the frequency (days per week) and duration (hours per day) they engaged in moderate-intensity PA, vigorous-intensity PA, and walking (not including for transportation to/from work) for at least 10 min at a time as a part of their work. Similarly, for transportation-related PA, the participants were asked to report how many days per week and hours per day they spent walking and bicycling to/from work, given the trip was at least 10 min. The IPAQ has been previously shown to be a reliable and valid instrument for assessing PA [17]. Two items from the IPAQ were used to assess the usual time spent sitting during the last 7 days in hours per day.

The IPAQ was scored to calculate a comparable summary estimate (total minutes per week) to the other PA measures. First, the total number of hours per week of each of the questions was calculated by taking the product of the reported number of days per week and the total time (hours) spent per day engaging in each of the activity intensities. Then, the total number of hours per week was multiplied by 60 to determine the total number of minutes per week of each of the questions. Finally, the sum of the total number of minutes per week for each of the PA intensity was calculated; walking for work and transport and bicycling for transport were considered moderate-intensity PAs [18]. Logically, activities described as vigorous in intensity (eg, heavy lifting, digging, and climbing stairs) and moderate in intensity (eg, carrying light loads) were categorized as such. The IPAQ MVPA summary estimate is the sum of the moderate- and vigorous-intensity PAs calculated by the respective questions.

Self-reported sedentary behavior, separate from what was queried in the IPAQ, was assessed with 8-items that inquire about the time, in hours per day, spent watching television and using the computer during the week and on the weekends [19].

#### Device-Based (Accelerometer) Physical Activity and Sedentary Behavior Measures

Device-based PA was assessed using the ActiGraph GT3X accelerometer (ActiGraph). The ActiGraph is a small (3.8 x 3.7 x 1.8 cm) triaxial piezoelectric accelerometer that is typically worn at the waist. Data outputs from the ActiGraph accelerometer are activity counts, which quantify the amplitude and frequency of detected accelerations. Activity counts are summed over a researcher-specified time interval (ie, epoch). In this study, a 60-second epoch was reported. The sum of the activity counts in a given epoch is related to activity intensity and can be categorized on the basis of validated activity count cut points [20]. Technical specifications, as well as the reliability and validity of the ActiGraph have been described previously [20,21]. Participants were asked to wear the ActiGraph (dominant hip) everyday, during all waking hours. After the 7-day study period, the participants returned the accelerometer to study staff. The data from the accelerometer were downloaded and screened for wear time using methods reported by Troiano et al [22]. Briefly, device nonwear time was defined as 60 consecutive min of 0 counts, with an allowance for 1to 2 min of detected counts between 0 and 100. Wear time was determined by subtracting derived nonwear time from 24 hours [22,23]. A minimum of 10 hours of wear time per day was required for data to be considered for further use in calculating daily estimates of PA/inactivity. Weekly summary estimates were computed by averaging daily estimates across the total number of days worn for participants with ≥4 days (out of 7 days) with ≥10 hours per day of wear time.

### Statistical Analysis

First, descriptive univariate analyses were conducted on measured parameters, and all continuous estimates were assessed for normality using histograms and Shapiro-Wilk tests. Continuous estimates were reported as means with standard deviations (SD) and medians with 25th and 75th percentiles, depending on normality; frequencies and percentages were reported for categorical variables. Next, the linear associations between and within self-reported (ie, EMA, BRFSS, and IPAQ) and device-based estimates were computed using Spearman rank-order correlation coefficients. Significance of the Spearman correlation coefficient was tested using Holm’s sequential Bonferroni adjustment. Then, the agreement between the inverse hyperbolic tangent transformed (*z*-transformation) self-reported and device-based estimates were assessed using Lin’s concordance correlation (LCC) coefficients with 95% CIs. Generally, LCCs are considered poor if they are less than .90 [24]. A visual representation of agreement of MVPA estimates was also obtained via Bland-Altman plots of the log transformed mean of MVPA from the self-report instruments and device-based assessment (x-axis) with difference in log transformed MVPA from the self-report instruments and device-based assessment (y-axis). All analyses were completed on a complete case analysis basis. The alpha level denoting statistical significance for all tests was set at .05. All statistical analyses were conducted via Stata/IC version 13.1 (StataCorp LP).

## Results

### Participant Characteristics

As shown in Table 1, the mean age of participants was 43.3 years. Of the 238 participants, 160 (67.2%) were female, 123 (51.7%) were black or African American, and 178 (74.8%) were overweight or obese. Most participants reported at least some college education (173/238, 72.7%) and 57.1% (136/238) were employed full-time. Almost two-thirds (150/238, 63.0%) of the participants were classified as low income. Overall, participants completed 92.9% (95% CI 0.915-0.941) of 1666 possible EMA daily dairy assessments via mobile phone over the 7-day study period. Additionally, 79.8% (190/238; 95% CI 0.742-0.847) participants wore the accelerometer for at least 10 hours on at least 4 days over the 7-day study period.

### Physical Activity and Sedentary Behavior

The median (25th, 75th percentiles) and the mean (SD) duration of time (minutes per week) spent in each of the activity intensities as measured by self-report and accelerometer are shown in Table 2. Tests for normality revealed that PA estimates were all non-normal and positively skewed, and all sedentary time estimates, except for those derived from accelerometer, were non-normal and positively skewed as well. Across all self-reported sedentary measures, participants underreported the time spent sedentary when compared with accelerometer-determined sedentary time. On the basis of accelerometer data, participants spent 3400.8 (SD 864.0) min per week sedentary, a median (25th, 75th percentile) of 120.5 (65.0, 218.0) min per week in moderate-intensity PA and 0.0 (0.0, 2.0) in vigorous-intensity PA, which amounted to 121.5 (66.0, 225.0) min per week of MVPA.

### Correlations

Regarding self-reported time spent sedentary, the BRFSS and IPAQ sedentary estimates were shown to be highly correlated (*r*=.77, *P*<.001) whereas all other self-report measures presented acceptable associations (*r*=.35-.37, *P*<.01) with each other. When compared with accelerometer-determined sedentary time, only the corresponding EMA estimate was significantly correlated (*r*=.16, *P*<.05). Because of complete case analysis for missing data, the sample sizes for tests of correlations may differ (see Table 3 for the sample size for each statistical test). Tests for differences revealed that participants who failed to adhere to the EMA and accelerometry protocols did not differ significantly (*P*>.05) from those who adhered to the protocols, based on age, race, BMI, employment status, and income.

With regard to the moderate-intensity PA summary estimates, participants over-reported the time spent in moderate-intensity PA when measured using BRFSS and IPAQ. Among the traditional self-report estimates only, the instruments displayed acceptable correlations (*r*=.33-.44, *P*<.01). Although all the traditional self-report estimates were significantly correlated to the accelerometer estimate (*r*=.17-.29, *P*<.05), the EMA estimate performed the best with a correlation coefficient of .29 (*P*<.01).

For the estimates reflecting vigorous-intensity PA, all of the traditional self-report and EMA measures overestimated the amount of time spent in vigorous-intensity activity when compared with accelerometer-derived estimates. Within the traditional self-report and EMA measures, correlations were low (.18-.29) but significant (*P*<.01). When compared with accelerometer-derived vigorous-intensity PA, none of the traditional self-report or EMA measures were significantly correlated (*r*=−.13 to .10, *P*>.05).

Finally, for MVPA, participants over-reported the amount of time spent in MVPA when compared with accelerometer estimates. The traditional self-report and EMA measures presented acceptable correlations within each other (*r*=.35-.46, *P*<.01). When compared with accelerometer-derived estimates of MVPA, the EMA estimate displayed an acceptable correlation (*r*=.31, *P*<.01) whereas the correlations with traditional self-report measures were low but significant (*r*=.17-.20, *P*<.05).

**Table 1:** Characteristics of Pathways between Socioeconomic Status and Behavioral Cancer Risk Factors Study participants, 2012.

Characteristic		Total (N=238)
**Age in years, mean (SD^a^)**		43.4 (13.1)
**Sex, n (%)**		
	Male	78 (32.8)
	Female	160 (67.2)
**Ethnicity/race, n (%)**		
	White	73 (30.7)
	Black or African American	123 (51.7)
	Asian	8 (3.4)
	American Indian, Alaska Native	2 (0.8)
	Hispanic/Latino	28 (11.8)
	More than one race	4 (1.7)
**Body mass index^b^**		
	Mean (SD)	30.6 (7.8)
	Underweight, n (%)	3 (1.3)
	Healthy weight, n (%)	57 (24.0)
	Overweight, n (%)	70 (29.4)
	Obese, n (%)	108 (45.4)
**Employment status, n (%)**		
	Employed (full-time or part-time)	136 (57.1)
	Unemployed	41 (17.2)
	Other^c^	61 (25.6)
**Educational attainment, n (%)**		
	No high school or GED^d^	22 (9.2)
	High school or GED	43 (18.1)
	Some college or more	173 (72.7)
**Household income, n (%)**		
	Below 2011 Federal Poverty Threshold^e^	150 (63.0)

^a^SD: standard deviation.

^b^Body mass index calculated as the reported weight in kilograms/(height in meters)^2^and classified based on World Health Organization cut points for adults.

^c^Other occupational statuses include homemaker-not employed, student-not employed, retired-not employed, unable to work or disabled, other.

^d^GED: general education development.

^e^Household income≤100% Federal Poverty Threshold (FPT) to 199% FPT.

**Table 2:** Duration of time spent in each physical activity intensity range as determined by five measurement devices among Pathways between Socioeconomic Status and Behavioral Cancer Risk Factors Study participants, 2012.

Intensity	Measurement device	Mean (SD^a^) (minutes/week)	Percentiles (minutes/week)		
			25th	50th	75th
Sedentary	EMA^b^	2320.2 (1998.8)	1080.0	1980.0	3120.0
	Self-report	2862.3 (1676.4)	1680.0	2511.5	3655.0
	IPAQ^c^	2897.1 (1559.6)	1800.0	2640.0	3720.0
	Accelerometer	3400.8 (864.0)	2813.0	3502.5	3996.0
Moderate	EMA	141.5 (98.4)	71.5	111.5	201.5
	BRFSS^d^	414.7 (657.6)	75.0	213.5	450.0
	IPAQ	754.7 (1312.9)	75.0	232.0	840.0
	Accelerometer	155.8 (139.0)	65.0	120.5	218.0
Vigorous	EMA	92.4 (82.5)	31.5	64.3	127.0
	BRFSS	212.9 (431.8)	0.0	63.5	240.0
	IPAQ	147.6 (429.6)	0.0	0.0	10.0
	Accelerometer	7.4 (25.7)	0.0	0.0	2.0
MVPA^e^	EMA	233.9 (163.3)	113.0	183.0	314.0
	BRFSS	627.6 (1592.4)	120.0	360.0	765.0
	IPAQ	902.3 (1592.4)	75.0	246.0	924.0
	Accelerometer	163.2 (151.4)	66.0	121.5	225.0

^a^SD: standard deviation.

^b^EMA: ecological momentary assessment.

^c^IPAQ: International Physical Activity Questionnaire.

^d^BRFSS: Behavioral Risk Factor Surveillance System.

^e^MVPA: moderate- to vigorous-intensity physical activity.

**Table 3:** Spearman correlation coefficients between five physical activity measurement devices among Pathways between Socioeconomic Status and Behavioral Cancer Risk Factors Study participants, 2012.

Intensity	Measurement device	Spearman correlation coefficients^a^					
		Accelerometer^b^		IPAQ		BRFSS	
		ρ (*P* value)	n	ρ (*P* value)	n	ρ (*P* value)	n
Sedentary	EMA^d^	.16 (.03)	168	.37 (<.001)	227	.35 (<.001)^c^	227
	Self-report	.07 (.33)	190	.77 (<.001)	238		
	IPAQ^e^	.08 (.27)	190				
Moderate	EMA	.29 (.001)	168	.42 (<.001)	206	.33 (<.001)	206
	BRFSS^f^	.17 (.02)	190	.44 (<.001)	238		
	IPAQ	.24 (.001)	190				
Vigorous	EMA	.09 (.26)	168	.28 (<.001)	206	.29 (<.001)	206
	BRFSS	.10 (.15)	190	.18 (.006)	238		
	IPAQ	−.13 (.08)	190				
MVPA^g^	EMA	.31 (<.001)	168	.42 (<.001)	206	.35 (<.001)	206
	BRFSS	.17 (.02)	190	.46 (<.001)	238		
	IPAQ	.20 (.006)	190				

^a^Significance tested using Holm’s sequential Bonferroni adjustment.

^b^Accelerometer data were classified as sedentary, moderate, or vigorous using Freedson’s cut points.

^c^This statistic indicates the Spearman correlation coefficient and *P* value for EMA and the self-reported measure of sedentary behavior from Healy et al (2011) [19].

^d^EMA: ecological momentary assessment.

^e^IPAQ: International Physical Activity Questionnaire.

^f^BRFSS: Behavioral Risk Factor Surveillance System.

^g^MVPA: moderate- to vigorous-intensity physical activity.

### Agreement

Agreement between the traditional self-report and EMA measures and device-based PA and sedentary behavior measures are shown in Table 4. Considering the time spent sedentary, when compared with accelerometer-derived estimates, each of the self-reported estimates showed no statistically significant agreement. With regard to the moderate-intensity PA estimates, BRFSS and IPAQ displayed low nonsignificant agreement to the accelerometer-derived estimates ([LCC=.12, 95% CI −0.02 to 0.26] and [LCC=.04, 95% CI −0.10 to 0.19], respectively) and low accuracy (bias correction factor [BCF]=0.42 and BCF=0.19, respectively). Only EMA estimates of moderate-intensity PA presented significant agreement (LCC=.32, 95% CI 0.19 to 0.46) and accuracy (BCF=0.94). Additionally, only EMA produced an acceptable measure of precision (*r*=.32, *P*<.001). The BRFSS and IPAQ estimates of vigorous-intensity PA had poor and statistically nonsignificant agreement to accelerometer estimates ([LCC=.02, 95% CI −0.12 to 0.16] and [LCC=−.05, 95% CI −0.19 to 0.09], respectively). EMA had poor agreement but a CI not containing a null result (LCC=.28, 95% CI 0.15 to 0.41) and a significant level of precision (*r*=.29, *P*<.001). None of the self-reports of vigorous-intensity activity had an accuracy estimate considered acceptable (BCF<0.90). Among the MVPA estimates, traditional self-report measures did not significantly agree with the accelerometer estimates, but there was agreement with EMA (LCC=.28, 95% CI 0.16 to 0.41). For MVPA, EMA was the only estimate to produce significant levels of precision (*r*=.32, *P*<.001) and an acceptable level of accuracy (BCF=0.90).

**Table 4:** Convergent validity of physical activity and sedentary behavior measurement devices as measured by Lin's concordance correlations, Pathways Between Socioeconomic Status and Behavioral Cancer Risk Factors Study, 2012.

Intensity	Measurement device	Accelerometer estimates^a^				
		LCC^b^	95% CI	Pearson *r*	*P* value	BCF^c^
Sedentary	EMA^d^	.07	−0.02 to 0.16	.12	.11	.61
	Self-report	.05	−0.06 to 0.16	.07	.35	.76
	IPAQ^e^	.06	−0.05 to 0.18	.08	.28	.80
Moderate	EMA	.32	0.19 to 0.46	.32	<.001	.94
	BRFSS^f^	.12	−0.02 to 0.26	.12	.09	.42
	IPAQ	.04	−0.10 to 0.19	.05	.54	.19
Vigorous	EMA	.28	0.15-0.41	.29	<.001	.21
	BRFSS	.02	−0.12 to 0.16	.02	.78	0.12
	IPAQ	−.05	−0.19 to 0.09	−.05	.50	.14
MVPA^g^	EMA	.28	0.16 to 0.41	.32	<.001	.90
	BRFSS	.08	−0.06 to 0.22	.09	.25	.30
	IPAQ	−.00	−0.03 to 0.03	−.002	.98	.18

^a^Accelerometer data were classified as moderate or vigorous using Freedson’s cut points.

^b^LCC: Lin’s concordance correlations.

^c^BCF: bias correction factor.

^d^EMA: ecological momentary assessment.

^e^IPAQ: International Physical Activity Questionnaire.

^f^BRFSS: Behavioral Risk Factor Surveillance System.

^g^MVPA: moderate- to vigorous-intensity physical activity.

**Figure 1:**
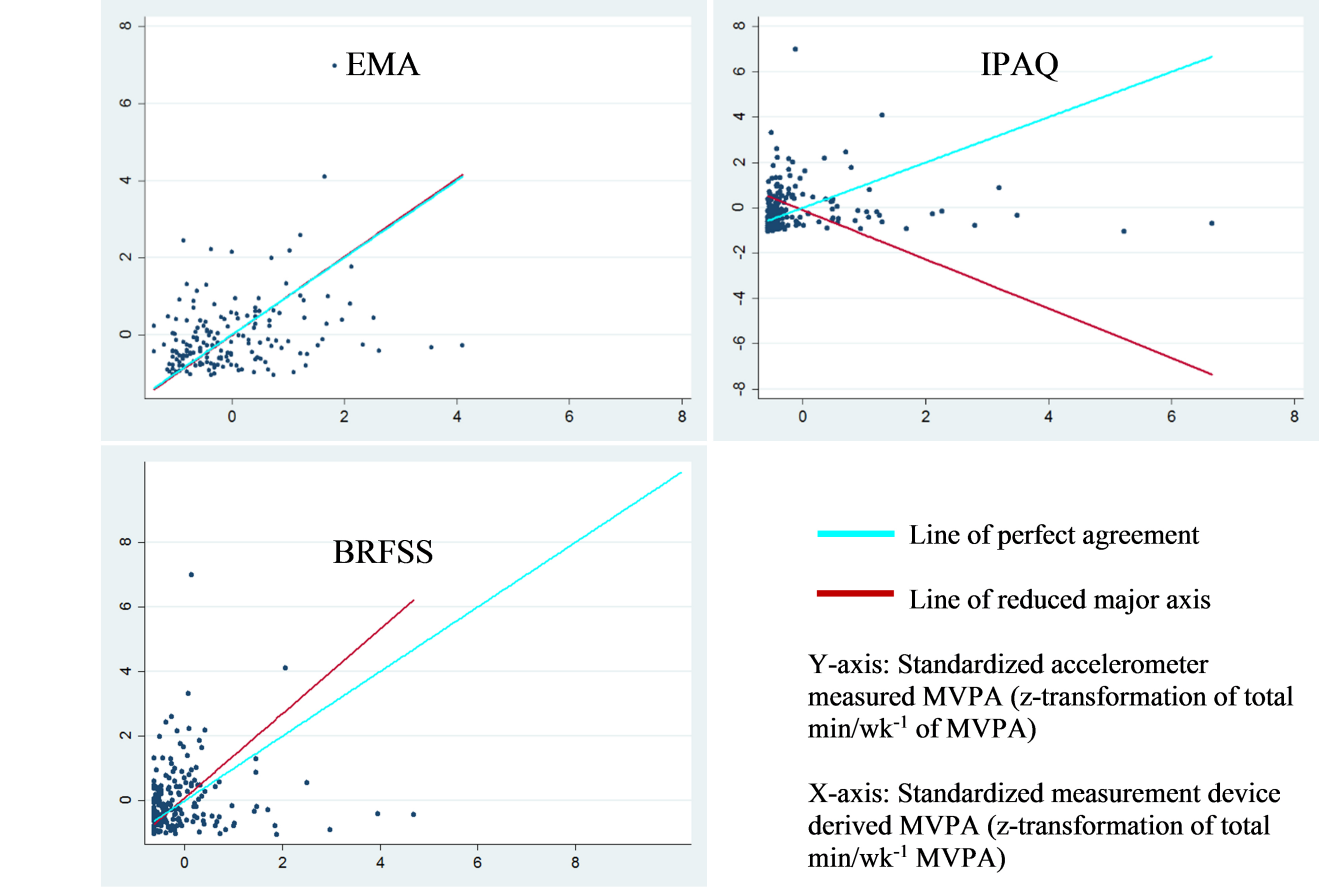
Lin concordance correlation plots of physical activity measurement devices to evaluate the agreement between the device and accelerometer in measuring moderate-, vigorous-, and moderate-to vigorous-intensity physical activity (MVPA).

**Figure 2:**
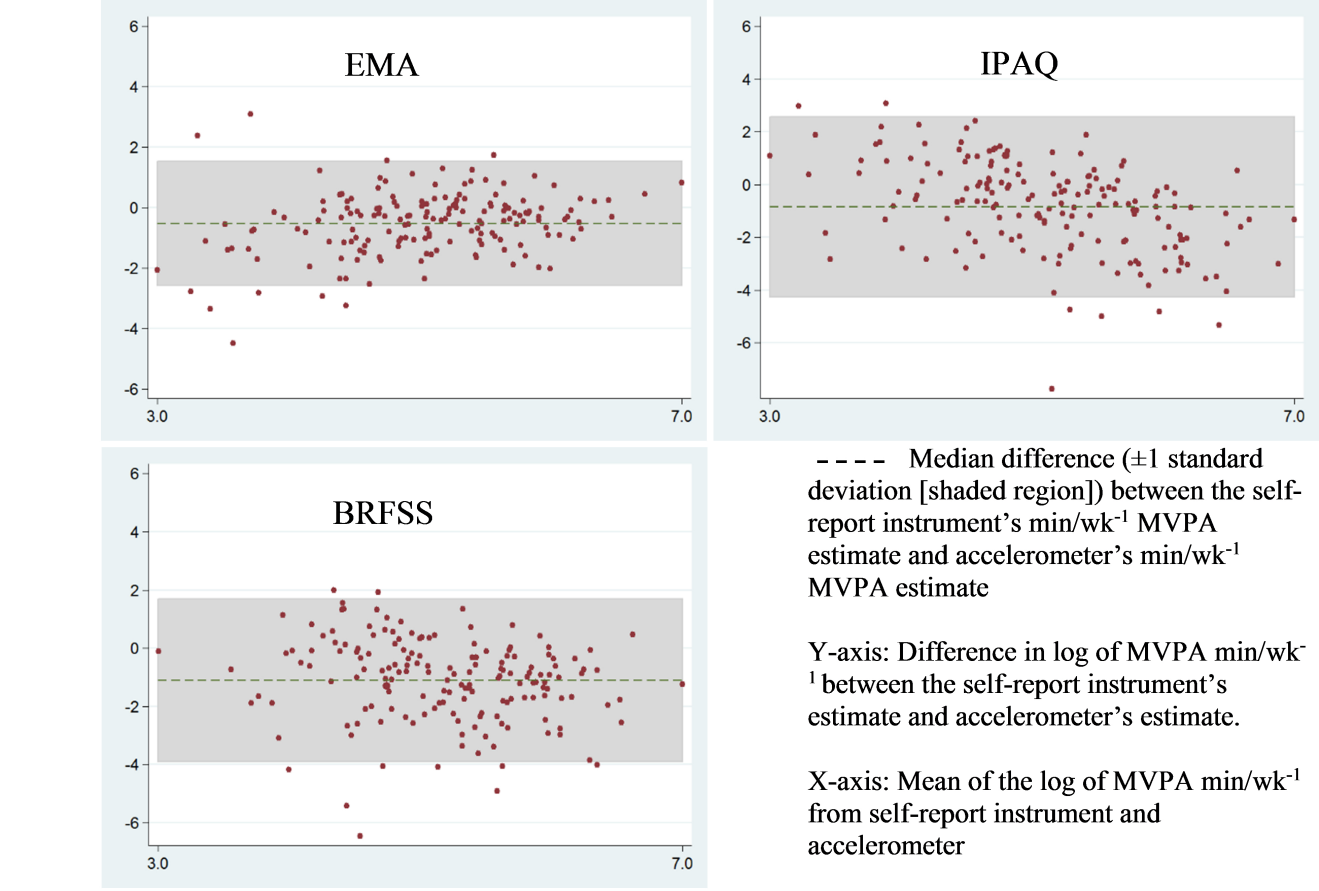
Bland-Altman plots (difference plots) of physical activity measurement devices to evaluate the agreement between the device and accelerometer in measuring moderate-, vigorous-, and moderate-to vigorous-intensity physical activity (MVPA).

Finally, Lin concordance correlation plots (Figure 1) and Bland-Altman plots (Figure 2) were also constructed to provide a visual representation of agreement between accelerometer-derived estimates of MVPA and the self-report measures. There does not appear to be a trend or pattern of the data points for any of the plots. For all the plots, the majority of the data points appear to be within the limits of agreement, yet none of the plots have all points within the limits of agreement. However, the limits of agreement do appear to be narrower for EMA than the other traditional self-report measures.

## Discussion

### Principal Findings

The results of this study indicated that EMA performed better than other forms of PA self-report measures when compared against accelerometry. The BRFSS and IPAQ measures indicated over-reporting of an average of 464.4 and 902.3 min per week of MVPA, respectively, compared with EMA over-reporting MVPA by an average of 70.7 min per week. Similarly, EMA performed better than the other self-report measures in the areas of correlation (moderate-intensity PA and MVPA) and agreement (moderate- and vigorous-intensity, and MVPA). Additionally, the strong participant compliance to the EMA (93%) protocol further supports the feasibility of EMA as a method to assess PA and sedentary behaviors. This finding, considering the diverse sample in this analysis, underscores the ease-of-use of mobile phones in assessing PA and other health-related behaviors.

These findings align with other studies that have found EMA to be a valid tool to estimate PA when compared with an objective assessment device. Dunton et al (2005) concluded that among adolescents, heart rates and accelerometer counts were significantly greater during EMA diary-reported PA than during non-PAs [25]. Similarly, Atenza and colleagues (2006) found in a pilot study that older adults’ reporting of minutes and frequency of moderate PA via EMA diary exhibited acceptable correlations to a standardized PA questionnaire for older adults (Community Healthy Activities Model Program for Seniors) [13]. Other studies from Dunton et al and Rofey et al have compared multiple randomly administered daily EMAs (rather than once per day diary) to accelerometer steps in children and adults and have concluded that EMA is a valid instrument to capture PA and sedentary behavior [12,26,27]. However, the aforementioned studies neither assessed the intensity of the PA nor the duration of the PA or sedentary time as was done in this study. Generally, previous studies and the findings of this study support the validity of EMA to measure PA and sedentary behavior.

Previous work in the measurement and evaluation of PA has primarily used correlation coefficients to assess reliability and validity. In this study, with the use of an accelerometer as a comparison measure, the calculated Spearman correlation coefficients support the convergent validity of EMA. The Spearman statistic presented for moderate-intensity PA, for example, is evidence that the EMA estimate of time spent engaging in moderate-intensity PA has a linear relationship, in direction and magnitude, with the accelerometer-derived estimate of time spent engaging in moderate-intensity PA. However, a drawback to this approach is that when only plotting the linear relationship between two estimates, the test is unable to detect departures from the plotted line that would indicate poor reproducibility or shifts in the data. For a more complete explanation on the drawbacks on the use of correlation coefficients to evaluate reproducibility and validity, see Lin (1989) [28]. Lin proposed the use of an index that combines measures of precision (Pearson correlation) and accuracy (bias correction factor [BCF]) to provide a superior measure of agreement by not relying on only one aspect of agreement but evaluating the agreement on the basis of both aspects [28]. The analyses in this study were able to determine that for sedentary behavior and PA, the EMA assessment performed the best in terms of agreement as measured by LCC when compared against an accelerometer. Although none of the LCC statistics for PA reached an acceptable threshold of .90, only EMA estimates for moderate-intensity PA, vigorous-intensity PA, and MVPA were statistically significant. Additionally, the estimates for moderate-intensity PA and MVPA can be considered to have acceptable correlations to the accelerometer [29]. Recently, Lin’s concordance correlations have been utilized to assess agreement in PA measurement [30] and have been further used in other areas of health behavior/outcomes research [31-33]. The LCC estimates for each estimate of self-reported PA was well below the acceptable threshold (.90) of agreement. It should be noted that the self-reports of PA are intended to measure the behavior of PA whereas accelerometry measures ambulatory movement. Furthermore, accelerometry is not considered the gold-standard assessment of PA behavior but rather an estimate of ambulatory movement that is less prone to bias [4].

This study compared estimates of PA derived from accelerometry with various self-report measures (ie, BRFSS, IPAQ, and EMA). These are estimates of total PA, whereas each of the self-report tools measures various different domains of PA. There are four proposed domains of PA that make up an individual’s total PA: (1) leisure-time PA, (2) transit-related PA, (3) occupation-related PA, and (4) household/domestic-related PA [34]. Each of the self-report PA assessment tools utilized in this study measured different domains of PA: EMA, leisure-time PA; IPAQ, occupation- and transportation-related PA; and BRFSS, leisure-time PA. None of the tools assessed domestic/household-related PA. Our results indicate that participants tended to overestimate their PA, regardless of domain. This finding aligns with many previous studies that indicate the possibility of social desirability bias or recall bias requiring complex computations, which may be inflating the self-reported PA estimates [1,35,36].

### Strengths and Limitations

This study has strengths and limitations. The population sample primarily included non-white and low-income urban-dwelling adults. This is a subgroup that is less frequently included in validation studies; however, much of the focus in PA research is directed at this subgroup for their disparate prevalence of many of the illnesses related to physical inactivity. Participants were enrolled as a convenience sample, and they may have inherent volunteer bias. Additionally, the PA assessment devices (self-report surveys and device-based) capture different aspects of PA (self-report instruments: PA behavior; device-based: PA–related movement) while attempting to compare one against the other as a means to validate. Finally, the time when PA was assessed differed across instruments (BRFSS, “usual week”; IPAQ, “past 7 days”; EMA, “past 24 hours”; accelerometer, as it occurred). Previous reviews have found that self-report questionnaires asking about the previous week showed slightly higher correlation than those asking about a usual week [37]. Additionally, the accelerometer and EMA were measuring PA during the same 7-day period, which could account for the higher correlations and measures of agreement. Though these data are not temporally matched, the validity of the IPAQ versus the accelerometer was determined within a relatively tight, 2-week window. Intuitively, one would not expect PA behavior to change drastically within a 2-week period unless there was a rare event (eg, sudden illness and vacation). Previous studies have shown that PA does not significantly differ within 2-week windows [38]. Though this aspect was not assessed in this study, we anticipate that very few (if any) participants experienced an event that would result in meaningful differences in PA.

### Conclusions

In conclusion, direct “real-time” assessment of PA can remove many of the inherent biases found in self-reporting of PA that can result in overestimation. However, device-based PA assessment can require expensive equipment and lacks contextual information on the PA. The EMA is a proposed self-report measure of PA that may minimize bias by reducing recall time while also providing context. This study suggests that mobile EMA is a practical tool for assessing PA behavior. Overall, mobile EMA performed better than the other more traditional forms of self-report of PA assessment, indicating its potential ability to overcome bias. Future research should focus on a larger, more representative sample and longer EMA protocols to determine if the compliance to the protocols remains after a certain period of time.

## Abbreviations

BCF: bias correction factor
BMI: body mass index
BRFSS: Behavioral Risk Factor Surveillance System
EMA: ecological momentary assessment
IPAQ: International Physical Activity Questionnaire
LCC: Lin’s concordance correlation
MVPA: moderate-, vigorous-, and moderate-to vigorous-intensity physical activity
PATHS: Pathways between Socioeconomic Status and Behavioral Cancer Risk Factors Study
PA: physical activity
SD: standard deviation
